# Increasing access to specialty care for rare diseases: a case study using a foundation sponsored clinic network for patients with neurofibromatosis 1, neurofibromatosis 2, and schwannomatosis

**DOI:** 10.1186/s12913-018-3471-5

**Published:** 2018-08-29

**Authors:** Vanessa L. Merker, Annie Dai, Heather B. Radtke, Pamela Knight, Justin T. Jordan, Scott R. Plotkin

**Affiliations:** 1 Department of Neurology and Cancer Center, Massachusetts General Hospital, 55 Fruit Street, Boston, MA 02114 USA; 2 Department of Health Policy and Management, Boston University School of Public Health, 715 Albany St, Boston, MA 02118 USA; 3 Harvard College, Cambridge, MA 02138 USA; 4 Children’s Tumor Foundation, 120 Wall Street, New York, NY 10005 USA; 5 Medical College of Wisconsin, 9000 W. Wisconsin Avenue, Milwaukee, WI 53226 USA

**Keywords:** Neurofibromatosis, Schwannomatosis, Rare diseases, Health service research, Health services accessibility, Healthcare disparities

## Abstract

**Background:**

Our primary aim was to assess the ability of a non-profit foundation-sponsored clinic network to facilitate access to specialized care for patients with neurofibromatoses (NF), a group of neurogenetic disorders including NF1, NF2, and schwannomatosis (SWN). Our secondary aim was to identify how our findings in NF could be applied more broadly to other rare diseases.

**Methods:**

We retrospectively reviewed aggregate data on patient volume reported by specialty NF clinics in a nonprofit network from 2008 to 2015. We classified clinics as high or low volume for disease type (NF1 and NF2/schwannomatosis) and pediatric/adult care. We compared clinic-level data to self-reported patient-level data from a large online patient registry.

**Results:**

Between 2008 and 2015, the number of certified NF clinics grew from 32 to 50, and annual patient volume rose from 6776 to 10,245 patients (13% of the total estimated U.S. NF patient population). For patient registry participants (*n* = 4476), the median driving distance to the nearest network clinic was 51.3 miles. Driving distances to reach high-volume centers were elevated for adults compared to children (295.8 vs. 67.9 miles), and schwannomatosis and NF2 patients compared to NF1 patients (310.9 vs. 368.1 vs. 161.7 miles). Of registry participants reporting their location of care (*n* = 2271), only 43.2% received care in a network specialty clinic, with especially low rates of attendance in the Southwest and Far West.

**Conclusions:**

While the number of certified NF clinics and volume of patients seen in these clinics has increased, many NF patients still do not attend specialty clinics and/or travel a significant distance for care. Geographic access to care is more limited for adults, patients with rarer conditions, and patients in the Western U.S. Potential measures to improve access to specialty care for people living with NF and other rare diseases are discussed.

## Background

In the United States, rare diseases are defined as conditions which affect less than 200,000 Americans. Patients with rare diseases may have difficulty identifying and accessing specialized clinical care. General clinicians are likely to be unfamiliar with most of the more than 6500 identified rare diseases, and a limited number of health care professionals are likely to specialize in each rare disorder. Lack of appropriate disease-specific care may contribute to delays and inaccuracies in diagnosis and treatment, which can cause substantial burden to the approximately 25 million people in the United States who are affected by a rare disease [1].

Having a certified, publicized network of specialty clinics may help patients with rare diseases access specialized disease care. Multiple rare disease foundations in the United States have established networks of accredited specialty centers, including the Cystic Fibrosis Foundation, the Von Hippel-Lindau Alliance, and the Charcot-Marie-Tooth Association. In 2007, a specialty network of neurofibromatoses (NF) clinics was created by the Children’s Tumor Foundation (CTF), a nonprofit research and patient advocacy organization. The neurofibromatoses are a group of three distinct neurogenetic disorders - neurofibromatosis type 1 (NF1), neurofibromatosis type 2 (NF2) and schwannomatosis (SWN). All three disorders cause a predisposition towards developing multiple nerve sheath tumors, with neurofibromas occurring in patients with NF1 and schwannomas occurring in patients with NF2 and SWN [2–4]. The neurofibromatoses are rare diseases, with estimated prevalences of 1 in 4560 for NF1 [5], 1 in 56,161 for NF2 [5], and 1 in 126,315 for SWN [6].

It is currently unknown how many NF patients in the United States receive specialized NF care or how accessible this care is. We evaluated the CTF-sponsored network of NF specialty clinics to 1) describe the current availability and utilization of specialty NF services in the U.S. and 2) assess for any potential disparities in access based on patient’s age, disease type, or region of residence. Access to health care is a multi-dimensional construct that has been conceptualized in many different ways; in this study we assess what Levesque et al. referred to as the “availability” of specialty NF services by looking at the number and locations of NF network clinics over time [7]. Given the lack of any publicly available, comprehensive listings of NF clinics, we use the clinic network as a proxy measure for the overall availability of specialty NF care in the U.S. In addition, we used geographic information systems to calculate driving distances between NF patients’ homes and network clinics, thus quantifying patients’ “actual geographical access” [8]. In doing so, we hoped to identify any patient subgroups with reduced access to care, and offer potential strategies for improvement that could be applied to clinic networks for NF and other rare diseases.

## Methods

### Data collection

We studied a network of neurofibromatosis clinics based in U.S. academic medical centers and certified by the Children’s Tumor Foundation. Any U.S. based NF clinic may apply to join the network; applications are reviewed by a group of medical professionals and foundation staff using a range of factors including demonstrated clinical expertise in NF, availability of multidisciplinary care, patient volume, and involvement in NF research. While this voluntary network does not include every dedicated NF clinic in the U.S., the foundation has made sustained efforts over the last decade to include as many clinics as possible. These efforts include promotion of the network at neurofibromatosis, pediatric neurology, and genetics conferences, as well as personal outreach to neurologists and geneticists in areas not currently represented in the NFCN.

All network clinics submit annual reports to the foundation detailing clinic activity in order to maintain status as a certified NF clinic. We retrospectively reviewed annual reports submitted by each network clinic from the years 2008 (the conclusion of the first year in which the network operated) to 2015, excluding 2011 because data was unavailable from the foundation for this year. We extracted data reported by staff members from each clinic for geographic location, clinic director specialty, and patient volume. Patient volume was requested for the time period ranging from July 1st of the previous year to June 30th of the current year, and was reported in aggregate by patient diagnosis (NF1, NF2, schwannomatosis, or other). Clinics also provided the number or percent of their yearly patient volume that was pediatric (age < 18) and adult (age ≥ 18).

Following proposed criteria currently under consideration by the foundation for use in defining NF Centers of Excellence, we classified clinics as having high volume in NF1 if they saw at least 200 NF1 patients per year and as high volume in NF2/SWN if they saw at least 20 NF2/SWN patients per year. No volume criteria were proposed regarding patient age; to examine differences in access by age, we arbitrarily classified clinics as high-volume in pediatric and/or adult care if they saw at least 100 patients age < 18 years or age ≥ 18 years, respectively.

We also reviewed prospectively-collected data from the CTF-sponsored NF Patient Registry, an online registry that contains self-reported (or parent-reported, in the case of patients under the age of 18) demographic and clinical data for over 7500 NF patients internationally [9]. For all living, U.S. based patients, we retrieved data as of October 14, 2015 on self-reported diagnosis, age, state of residence, home zip code, and clinic attended for NF care. Clinic attendance for NF care was added as a data field in the patient registry in October 2013, and was originally not a required variable, so data on this variable was not available for all participants. While some patients were recruited to the registry through advertisements posted at network clinics, efforts were also made to reach patients who do not attend network clinics using advertisements in foundation publications and on social media.

### Statistical analysis

Descriptive statistics were calculated based on total annual patient volume and volume by disease type and by age group. For all analyses, patient totals include only patients with NF1, NF2, or schwannomatosis (i.e. patients with “other” listed as diagnosis were excluded). For regional analyses, the United States was divided into eight regions (New England, Mideast, Great Lakes, Plains, Southeast, Southwest, Rocky Mountain, and Far West), as defined by the Bureau of Economic Analysis [10]. To estimate the total number of NF patients in the U.S., a U.S. population of 320,896,618 people was used (U.S. Census Bureau population estimate for July 1, 2015) [11]. Using the disease prevalence estimates noted above, this provided estimated U.S. patient populations of 70,372 people with NF1; 5713 people with NF2; and 2540 people with schwannomatosis. We also used state level population estimates for July 1, 2015 from the U.S. Census Bureau to calculate the percentage of the population living in each region and the number of NF patients estimated to live in each region [11].

The geographic distribution of network clinics and patient registry members was mapped based on zip code data using ArcGIS software (version 10.4.1; ESRI, Redlands, CA). Driving distance in miles between each registry member and the nearest network clinic was calculated using ArcGIS, both in the overall cohort and by corresponding clinic/patient subgroups (eg., driving distance between each NF1 patient and the nearest clinic with a high volume of NF1 patients, etc.) Analysis of driving distance excluded NF registry participants living in Alaska and Hawaii.

### Ethical consideration

This research was reviewed by was reviewed by the Partners Human Research Committee (Protocol 2015P001563) and determined to be exempt. The NF Registry protocol was approved by the Western Institutional Review Board in Protocol Number 20120455. All participants of the NF Registry (or their parent/legal guardians) provided online informed consent prior to entering any data into the NF Registry; informed consent includes sharing of non-identifiable information with other researchers.

## Results

### Overall characteristics

**Fig. 1:**
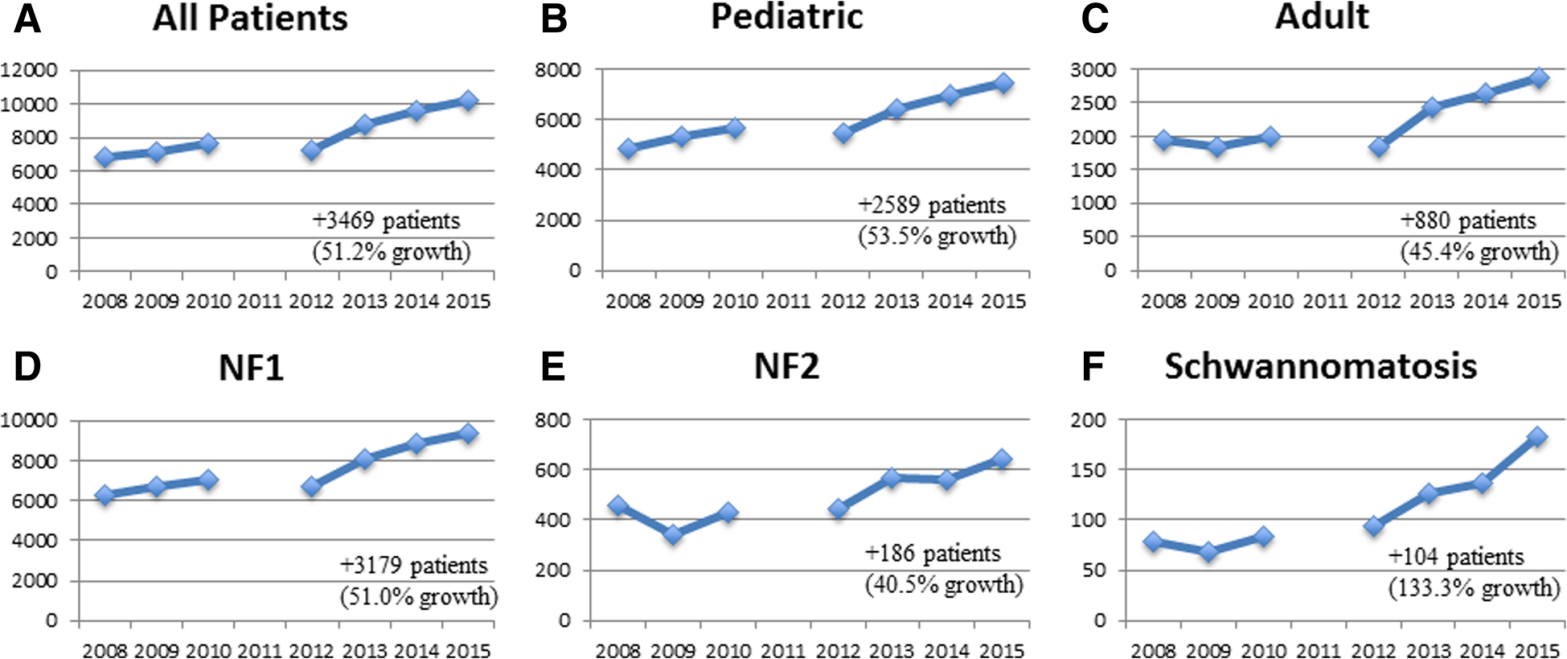
Growth of NF patient volume in a nonprofit foundation sponsored clinic network from 2008 to 2015. Note: Annual reports from 2011 were unavailable for analysis

**Fig. 2:**
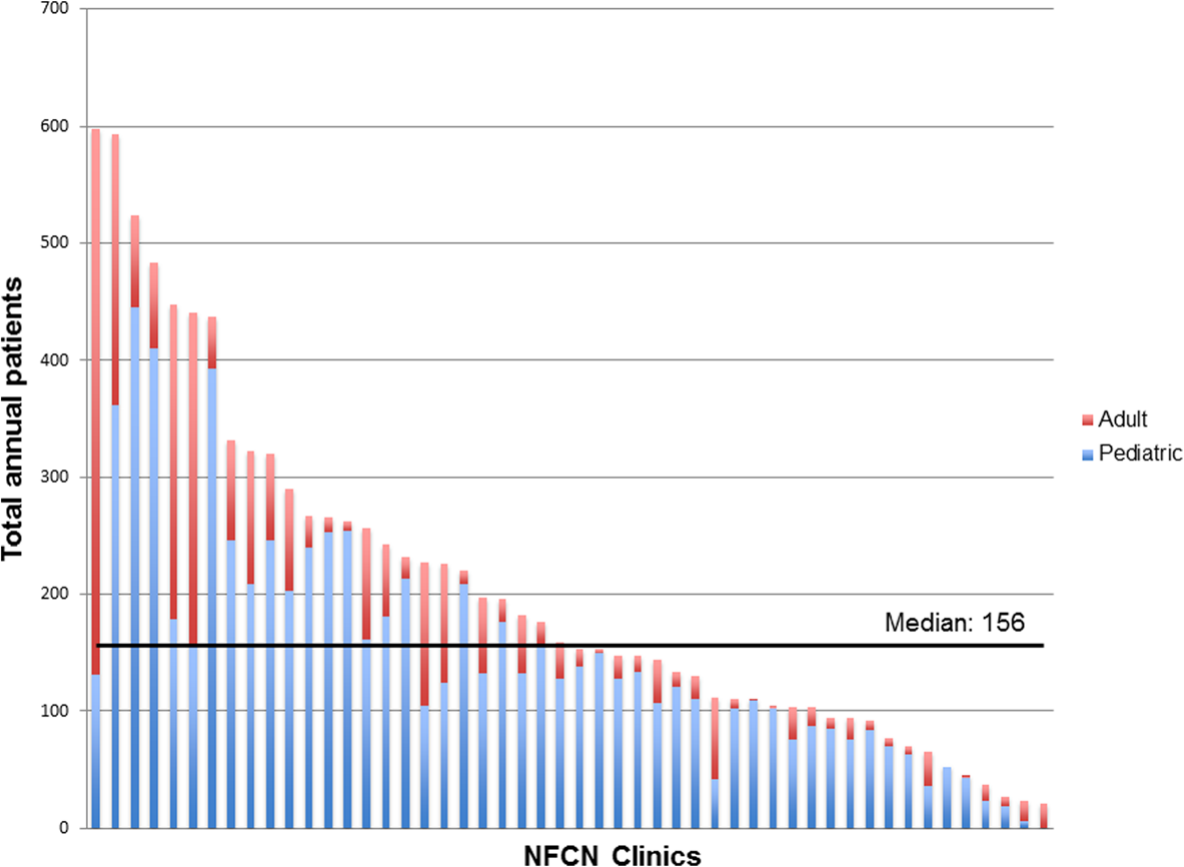
2015 NF patient volume (per clinic). Each bar represents the total number of NF patients seen between July 1st, 2014 and June 30th, 2015 at each clinic (*n* = 50) in the network. Across all clinics, 10,245 patients were seen: 7408 pediatric (age < 18 years) and 2837 adults (age ≥ 18 years)

The clinic network grew from 32 to 50 clinics between 2008 and 2015, and annual patient volume rose from 6776 to 10,245 patients per year (Fig. 1). The number of patients seen by each clinic, based on 2015 annual reports, is displayed in Fig. 2. Median annual clinic volume was 156 patients (25th percentile-75th percentile: 103–265 patients). Most patients seen across the network in 2015 had NF1 (*n* = 9418, 91.9%) and a minority had NF2 (*n* = 645, 6.3%) or schwannomatosis (*n* = 182, 1.8%). Compared with the estimated number of NF patients living in the U.S. (based on prevalence rates and U.S. population above), network clinics serve 13.0% (10,245/78,625) of the total U.S. NF patient population (NF1: 13.4%; NF2: 11.3%, SWN: 7.2%).

**Fig. 3:**
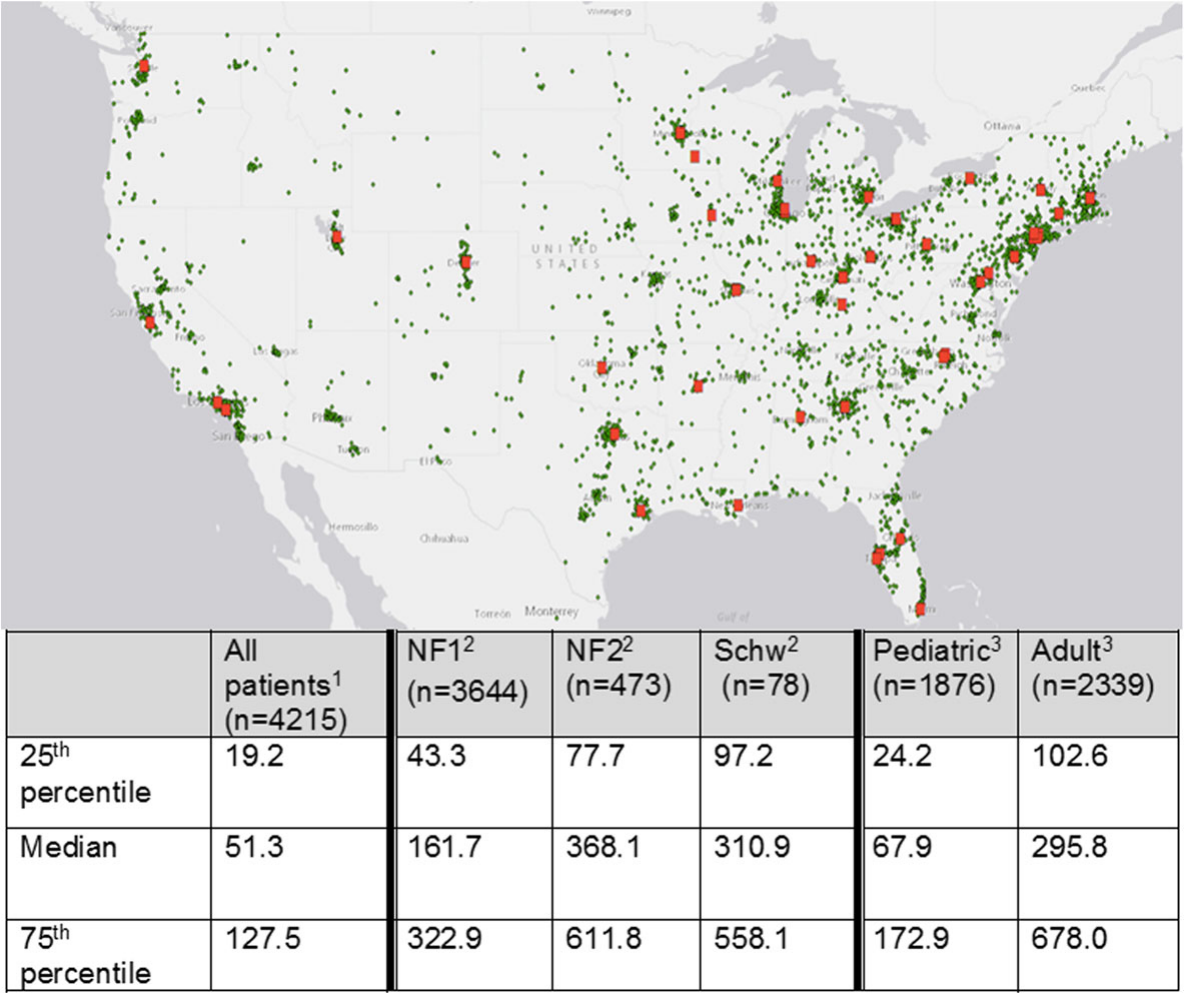
Geographic distribution of patient registry participants and driving distance (in miles) to network clinics. U.S. map showing the geographic location of NF patient registry participants (green dots) and network clinic locations (red dots). The table shows driving distance (in miles) from registry participants’ home zip code to ^1^nearest clinic; ^2^nearest clinic with high volume in their disorder (≥200 NF1 patients or ≥ 20 NF2/SWN patients); ^3^nearest clinic with high volume (≥100 patients) in their age group. Registry participants residing in Alaska and Hawaii were excluded

As of October 14, 2015, 4476 living U.S. patients with a confirmed diagnosis of NF1, NF2, or schwannomatosis were enrolled in the patient registry (Table 1). Of the 2271 registry patients who entered information about which clinic they attended, only 982 (43.2%) received care in a network clinic. The remaining 1289 individuals received NF-related care at NF clinic that was not certified by this foundation or from a non-NF specific practice (such as a general genetics clinic or a pediatrician). ArcGIS maps were created to compare the geographic distribution of patients in the online registry to the location of network clinics, as well as calculate the driving distance in miles between patients’ home zip codes and the nearest network clinic (Fig. 3). We found that NF patient registry participants would have to drive a median of 51.3 miles [interquartile range (IQR): 108.3 miles] to reach the nearest network clinic.

**Table 1:** Characteristics of U.S. based participants in an online registry of NF patients (as of October 2015)

	All patient registry participants	Participants attending an NF network clinic	Participants not attending an NF network clinic
Total	4476	982	1289
Age			
Pediatric	1980 (44.2%)	541 (55.1%)	493 (38.2%)
Adult	2496 (55.8%)	441 (44.9%)	796 (61.8%)
Disease Type			
NF1	3898 (87.1%)	844 (86.0%)	1093 (84.8%)
NF2	496 (11.1%)	119 (12.1%)	162 (12.6%)
Schwannomatosis	82 (1.8%)	19 (1.9%)	34 (2.6%)
Region			
New England	245 (5.5%)	80 (8.1%)	54 (4.2%)
Mid East	720 (16.1%)	224 (22.8%)	165 (12.8%)
Great Lakes	755 (16.9%)	185 (18.8%)	174 (13.5%)
Plains	383 (8.5%)	73 (7.4%)	97 (7.5%)
Southeast	1062 (23.7%)	241 (24.5%)	313 (24.3%)
Southwest	402 (9%)	74 (7.5%)	174 (13.5%)
Rocky Mountain	268 (6%)	46 (4.7%)	55 (4.3%)
Far West	641 (14.3%)	59 (6%)	257 (19.9%)

Data not broken out for subgroup of registry participants who did not indicate their location of NF care (*n* = 2205)

### Characteristics by disease type (2015)

Analysis of patient volume by disease type reveals four categories of clinics: high volume for NF1 (*n* = 13, 26%), high volume for NF2/SWN (*n* = 2, 4%), high volume for both NF1 and NF2/SWN (*n* = 6, 12%), or low-volume for both NF1 and NF2/SWN (*n* = 29, 58%). In total, nineteen clinics (38%) located across 16 states were high volume for NF1 and 8 clinics (16%) located across 7 states were high volume for NF2/SWN. There were no high-volume NF2/SWN clinics in the Far West or Rocky Mountain region. The median driving distance to the nearest high-volume clinic for NF2 patients and SWN patients was nearly double that of NF1 patients (368.1 [IQR: 534.1] and 310.9 miles [IQR: 460.9] vs. 161.7 miles [IQR: 279.6]) (Fig. 3).

### Characteristics by age group (2015)

The majority (48/54, 88.9%) of clinic directors participating in the network had pediatric training (four clinics had two equal co-directors). The majority of patients seen in network clinics were pediatric age (*n* = 7408, 72.3%). Thirty-five clinics (70%) were high-volume for pediatric patients whereas only 7 (14%) clinics were high-volume for adult patients. Twenty-nine clinics (58%) were located within dedicated children’s hospitals. Only 7 clinics (14%) saw more adult than pediatric patients per year. Among patient registry participants who reported the location of their NF care, 52.3% (541/1034) of children attended a network clinic, while only 35.7% (441/1237) of adults did so (Table 1). Adult patients would have to drive a median of 295.8 miles [IQR: 575.4] to reach a certified clinic with high-volume experience in their age group, while pediatric patients (and their families) drive a median of only 67.9 miles [IQR: 148.7] (Fig. 3).

### Characteristics by region (2015)

**Fig. 4:**
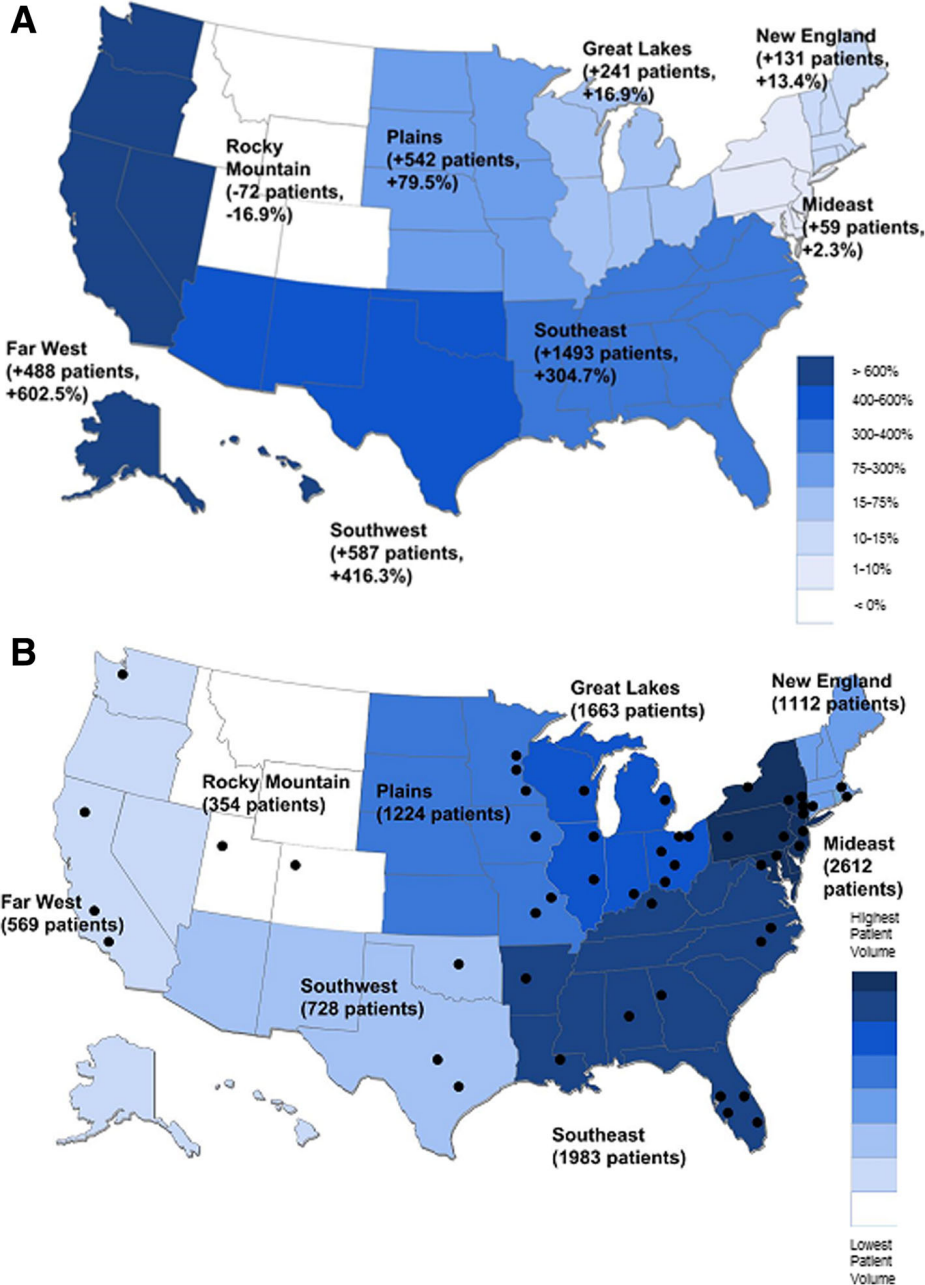
Aggregate Patient Volume by Region of Clinic Attended. Panel **a**: Growth in clinic network patient volume by region from 2008 to 2015, as determined by annual reports. Panel **b**: Total number of patient seen by region in 2015. Black dots represent network clinic locations in 2015

The distribution of clinics was not even across the United States. Twenty-three of fifty states (46%) lacked a certified clinic, while 12/50 states (24%) had more than one clinic. Most regions of the U.S. had an increase in patient volume between 2008 and 2015 (Fig. 4a). However, regions with the highest percentage growth (Far West and Southwest) still had a relatively low number of clinics and absolute number of patients as of 2015 (Fig. 4b). In 2015, clinics in the Mideast region reported the highest absolute patient volume (*n* = 2612, 25.5%) and clinics in the Rocky Mountain region had the lowest absolute patient volume (*n* = 354, 3.5%). However, relative to the overall population living in each region, clinics in New England had the highest relative patient volume (0.008%; 1112/14,710,229) and the Far West had lowest relative patient volume (0.001%, 569/55,225,488).

As a simplified measure of capacity for specialized NF care, we also estimated the number of NF patients living in each region in 2015 and compared this to the number of certified NF clinics in those regions. The Plains had the highest capacity for NF care (with 1 clinic for every 855 NF patients estimated to live in the region) while the Far West had the lowest capacity (with 1 clinic for every 3357 NF patients estimated to live in the region). [The remaining regions had an estimated 1085 (Mideast), 1136 (Great Lakes), 1192 (New England), 1424 (Rocky Mountains), 1814 (Southeast), and 3261 (Southwest) NF patients per network clinic.] Regions with the lowest capacity, such as the Southwest and Far West, had a higher percentage of NF Registry participants attending non-network clinics (Table 1).

## Discussion

Since 2008, efforts to expand access to specialty NF care through a network of certified specialty care clinics publicized by a rare disease non-profit foundation has resulted in a 56% increase in the number of certified NF clinics and a 51% increase in the number of NF patients served by the network. This growth was steady across all diagnosis groups (NF1, NF2, SWN), both age groups (pediatric and adult), and all but one geographic region. Growth in patient volume was especially dramatic in the Southeast, Southwest, and Far West regions, due both to the addition of newly certified clinics and an increase in patient volume at these clinics over time. Increased growth at specialty clinics may help improve the quality of care of patients with NF, as prior studies in the United Kingdom showed that NF2 patients treated at specialty clinics have a lower risk of mortality than patients treated at non-specialty centers [12]. In addition, recent guidelines for the care of patients with NF1 have recommended annual visits in specialized multidisciplinary clinics [13–15]. More broadly, research across a range of surgical conditions have shown that provision of care in specialized, high-volume centers can lead to better clinical outcomes [16].

Despite increased capacity within this clinic network, we estimate that only 13% of all U.S. NF patients are seen in certified clinics. While there were relatively substantial care resources available for pediatric care and for patients with NF1, fewer options existed for adults and for patients with NF2 or schwannomatosis. While NF2 and schwannomatosis are lower prevalence conditions than NF1, and so resources may be expected to be fewer for these patients, concerns for equity of access to healthcare necessitate attention to these disorders. Only 8 clinics were high-volume for NF2/SWN, and patients with NF2 or SWN subsequently had much larger median driving distance to reach a high volume center. To address low numbers of clinics, foundations could encourage clinicians specialized in specific disease features to expand into care for patients with rarer diseases (for example, helping surgeons experienced in sporadic schwannomas to provide care for NF2/SWN). Innovative funding and training programs, such as sponsored fellowship training opportunities at already established rare disease clinics, could assist in providing these clinicians with appropriate disease-specific knowledge in lower prevalence rare disorders such as NF2 and SWN.

Access to care for adults also remains a challenge in NF, and transitions from pediatric to adult care have been shown to be difficult across multiple rare disorders [17]. With the majority of network clinics being run by directors with pediatric training, and more than half of network clinics located in dedicated children’s hospitals, it is not surprising that the majority of NF adult registry participants do not attend a network clinic. Attendance at a high-volume clinic may be difficult for adults, as the median driving distance for adults was more than 4 times that of children. Further research into why adults may be unable to attend specialty rare disease clinics (for example, because nearby clinics do not accept adult patients) or choose not to attend these clinics (for example, because of personal preference or financial constraints) would be valuable in guiding a strategy for improving access to care. Research in Australia showed that many adults with NF1 did not receive regular specialist care due to a combination of these factors, including not knowing where to get care and not realizing the importance of continued monitoring in adulthood [18]. This indicates that educational awareness campaigns around the benefits of regular specialist care may increase the proportion of adults with NF1 attending specialty clinics. Possible interventions to increase capacity for adult care include capitalizing on the existing infrastructure at high-volume pediatric rare disease clinics to directly expand into adult care or encouraging pediatric clinics to partner with neighboring adult institutions to ease the transition from pediatric to adult care.

Expansion of clinic networks should also address regional disparities in care, which are apparent especially for patients living in the western regions of the United States. The Far West and Southwest have the lowest number of certified NF clinics per capita (on a population level), and most centers are located east of the Mississippi River, likely reflecting an uneven distribution of academic medical centers across the United States. Given that most rare disease clinics are housed within specialized academic medical centers, this geographic disparity likely extends across rare disorders. Potential measures to increase access to care for rare disease patients living in the West/Southwest include opening new clinics in these regions; increasing outreach into these regions using telemedicine; or by providing financial support to assist patients in traveling to specialized centers in other regions.

The creation of a network of NF clinics certified by a non-profit foundation can also serve as a learning opportunity for other rare disease groups. The network demonstrates the value of ongoing support and communication between medical clinics and a non-profit foundation, in terms of increasing rare disease patients’ access to high quality care. Annual reports from clinics can serve as a key data collection mechanism for quality metrics as they are developed. Patient registries, in which patients directly report what clinic they attend, can allow foundations to link patient reported measures of health status and satisfaction to individual clinics, in order to begin to assess the quality of clinics in the network (with the understanding that many factors beyond clinical quality also influence patient reported outcome measures.) A successful example of this model is The Cystic Fibrosis Foundation, which accredits clinics based on adherence to a comprehensive set of structural and process quality measures, and publicly reports outcome quality measures such as patients’ lung function and nutritional status at the clinic level [19, 20].

In this retrospective study, data are limited by their completeness and accuracy. Clinic staff members reported their own patient volume statistics and may not have reported accurately. When aggregating patient volume across all clinics, we may overestimate the number of NF patients seen in the network, as some patients may visit more than one clinic per year (for example, for second opinions or when transferring care).

While it is our belief that most specialized NF clinics in the U.S. belong to the clinic network, it is unknown how many non-certified NF clinics exist in the U.S. and how they may differ from certified clinics. In addition, access to the network is only a proxy measure for overall access to specialized NF care as some NF specialists may practice outside of established NF clinics. We categorized clinics as high-volume based on proposed criteria under consideration at the foundation and not on any pre-established markers for high quality care. While volume has been used as a proxy for high quality care in assessing specialized centers for surgery [21], patient access to high volume NF clinics may not be a sufficient proxy measure for access to high quality NF care.

For analyses of driving distance, approximately 5% of patient zip codes in the patient registry were not valid (*n* = 242) or were in Alaska or Hawaii (*n* = 19), and therefore were not utilized. In addition, since only approximately one-half of patient registry participants reported what clinic they attended, our analysis matched patients to the nearest network clinic, rather than the clinic each patient actually attended. This means our analysis of travel distance does not take into account patient preferences or other factors (like insurance coverage) that would lead patients to choose to attend clinics farther away from their home. Furthermore, since many network clinics recruit patients to the patient registry, the sample of patients who have joined the registry may be closer in geographic location to network clinics than the U.S. NF patient population as a whole. Finally, report of clinic attended was by a drop-down menu that could not distinguish between receipt of care from an NF clinic not accredited by this foundation and receipt of care from other locations (such as non-disease specific general practitioners). Recent changes to the patient registry that allow participants to report if they do not receive any specialized NF care should clarify this issue in the future.

## Conclusion

Overall, there have been marked improvements in access to specialized care for NF patients since 2008. But given that there are many NF patients in the U.S. who are not seen in specialized clinics and/or travel a significant distance for their care, a strategic plan to guide future network expansion would be valuable. To date, the network has grown organically based on the locations of practicing clinicians who were motivated to join the network. In the future, data on general population growth trends, data from clinic annual reports, and data from the patient registry can be combined to allow for precise targeting of expansion efforts to the regions and patient populations in the most need. For example, geographic information systems tools have been used to map multiple sclerosis specialty centers in the Veterans Health Administration and quantitatively assess the impact of adding new clinics on veteran’s access to care [22]. However, clinic availability is just one of multiple dimensions of healthcare access; other areas of both actual and perceived access to care will affect the number of NF patients who utilize services [8]. Efforts to improve access to NF specialty care will need to address a range of potential barriers to receiving specialty care (such as financial barriers or lack of information) in order to ensure that a greater proportion of NF patients receive high-quality, specialized NF care.

## Abbreviations

CTF: The Children’s Tumor Foundation
NF: Neurofibromatosis
NF1: Neurofibromatosis 1
NF2: Neurofibromatosis 2
SWN: Schwannomatosis
U.S.: United States
